# Genetic basis of index patients with familial hypercholesterolemia in Chinese population: mutation spectrum and genotype-phenotype correlation

**DOI:** 10.1186/s12944-018-0900-8

**Published:** 2018-11-06

**Authors:** Di Sun, Bing-Yang Zhou, Sha Li, Ning-Ling Sun, Qi Hua, Shu-Lin Wu, Yun-Shan Cao, Yuan-Lin Guo, Na-Qiong Wu, Cheng-Gang Zhu, Ying Gao, Chuan-Jue Cui, Geng Liu, Jian-Jun Li

**Affiliations:** 1 Division of Dyslipidemia, State Key Laboratory of Cardiovascular Disease, Fu Wai Hospital, National Center for Cardiovascular Disease, Chinese Academy of Medical Sciences and Peking Union Medical College, Beijing, 100037 China; 2 Department of Cardiology, Peking University People’s Hospital, Beijing, 100044 China; 3 Department of Cardiology, Xuanwu Hospital, Capital Medical University, Beijing, 100053 China; 4 Guangdong Cardiovascular Institute, Guangdong Provincial Key Laboratory of Clinical Pharmacology, Guangzhou, 510080 China; 5 Department of Cardiology, Gansu Provincial People’s Hospital, Lanzhou, 730000 Gansu China

**Keywords:** Familial hypercholesterolemia, Lipid, LDLR, APOB, PCSK9

## Abstract

**Background:**

Although there have been many reports in the genetics of familial hypercholesterolemia (FH) worldwide, studies in regard of Chinese population are lacking. In this multi-center study, we aim to characterize the genetic spectrum of FH in Chinese population, and examine the genotype-phenotype correlations in detail.

**Methods:**

A total of 285 unrelated index cases from China with clinical FH were consecutively recruited. Next-generation sequencing and bioinformatics tools were used for mutation detection of LDLR, APOB and PCSK9 genes and genetic analysis.

**Results:**

Overall, the detection rate is 51.9% (148/285) in the unrelated index cases with a total of 119 risk variants identified including 84 in the LDLR gene, 31 in APOB and 4 in PCSK9 gene. Twenty-eight variants were found in more than one individual and LDLR c.1448G > A (p. W483X) was most frequent one detected in 9 patients. Besides, we found 8 (7 LDLR and 1 APOB) novel variants referred as “pathogenic (or likely pathogenic) variants” according to in silico analysis. In the phenotype analysis, patients with LDLR null mutation had significantly higher LDL cholesterol level than LDLR defective and APOB/PCSK9 mutation carriers and those with no mutations (*p* < 0.001). Furthermore, 13 double heterozygotes, 16 compound heterozygotes and 5 true LDLR homozygotes were identified and the true LDLR homozygotes had the most severe phenotypes.

**Conclusions:**

The present study confirmed the heterogeneity of FH genetics in the largest Chinese cohort, which could replenish the knowledge of mutation spectrum and contribute to early screening and disease management.

## Background

Familial hypercholesterolemia (FH) is an inherited disorder mainly caused by the mutation of low-density lipoprotein receptor (LDLR) gene, apolipoprotein B (APOB) gene, or proprotein convertase subtilisin /kexin type 9 (PCSK9) gene in autosomal dominant pattern [1]. The extremely elevated level of low-density lipoprotein cholesterol (LDL-C) makes the patients with FH expose to high risk of premature atherosclerotic cardiovascular disease (ASCVD) [2]. According to the presence of one or two FH-causing alleles, patients with FH are divided into heterozygotes and homozygotes, whose prevalence was roughly 1 in 200–600 and 1 in 1,000,000 individuals respectively [3].

With the advancement of sequencing technique, the knowledge about molecular basis of FH has been expanded for recent years. Up to now, LDLR gene occupies the majority among the known causative mutations (90–95%), with APOB for 5–10% and PCSK9 for less than 3% [4]. Furthermore, rare proportion of FH is caused by the mutations in the LDLRAP1 gene with two pathological variants in autosomal recessive pattern [5]. To date, more genes including STAP1, APOE, and LIPA have been identified as possible FH-causing genes [6–8]. Among them, LDLR gene has acquired comprehensive investigations with an identification of more than 2900 variants in the Leiden Open Variation Database (LVOD) [9]. According to the functional changes, LDLR mutations have been classified into “null” mutations and “defective” mutations [10, 11].

The genotype-phenotype relationship of patients with FH showed high heterogeneity. Previous studies have found that 20–60% of subjects with phenotypic FH did not carry a causative mutation in LDLR, APOB, or PCSK9 genes, which could be explained by multiple small-effect common variants, mutations in unknown FH-associated genes or environmental effect [12–16]. Besides, the phenotypic severity exists on a continuum with a considerable overlap between heterozygous and homozygous FH (including double heterozygotes, compound heterozygotes and true homozygotes), though generally the mean LDL-C level increased as follows: LDLR-negative homozygotes > compound LDLR heterozygotes > LDLR-defective or LDLRAP1 homozygotes > APOB or PCSK9 homozygotes > double heterozygotes > heterozygous FH [17]. In spite of the heterogeneity, genetic diagnosis allows for early diagnosis and intervention for FH patients with the help of cascade screening, especially for the subjects who only meet borderline diagnostic criteria.

The Chinese population is comprised of multiple ethnic groups with distinguish regional distributions. Despite of previous reports in the mainland of China, Hongkong and Taiwan, there are still few systematic studies about molecular basis of Chinese FH population, especially focusing on unrelated index cases [18–20]. The aim of the current study is to further characterize the molecular basis of FH in an extended range with unrelated index cases form multiple centers, which could refine the genetic spectrum of FH in China and address the genotype-phenotype correlations.

## Materials and methods

### Study population

This study consecutively recruited 285 unrelated index cases of clinical FH, among which 279 were adults and 6 were children, from 2011 to 2017 in the division of dyslipidemia of Fuwai Hospital and four other centers. The adult patients were diagnosed with definite or probable FH according to Dutch Lipid Clinic Network (DLCN) criteria with a score ≥ 6. Children who had values of LDL-C above the 95th percentile according to age and gender and family history of high cholesterol and/or premature familial cardiovascular disease were considered to be FH [1].

Our study complied with the Declaration of Helsinki and was approved by the hospital’s ethical review board (Fu Wai Hospital & National Center for Cardiovascular Diseases, Beijing, China). Informed written consents were obtained from all the participants.

### Clinical and biochemical examination

Clinical data of each participant were collected by physicians and experienced nurses, including the prior lipid levels and use of lipid-lowering medications, family and personal history of dyslipidemia and coronary artery disease (CAD) as well as presence of tendon xanthoma and corneal arcus. In addition, a standardized physical examination consisted of height (m), weight (kg) and blood pressure was performed for each patient.

After at least 12-h fast, blood samples were collected from cubital vein for biochemical measurements. Serum total cholesterol (TC), triglyceride (TG), high-density lipoprotein cholesterol (HDL-C) and LDL-C were determined using an enzymatic assay with automatic biochemistry analyzer (Hitachi 7150, Tokyo, Japan). The concentrations of apolipoprotein A (apo A) and apolipoprotein (apo B) were measured by a turbidimetric immunoassay.

### Genetic sequencing

Peripheral blood samples were well preserved at − 80 °C until the genomic DNA extraction using a commercial DNA extraction kit (Tiangen Biotech, Beijing, China) with standard procedure. After the detection of DNA purity, the qualified samples were prepared for the targeted next generation sequencing (NGS) covering all the coding exons of LDLR (NM_000527), APOB (NM_000384) and PCSK9(NM_174936) genes. The hybridization reactions were carried out on a AB 2720 Thermal Cycler (Life Technologies Corporation, USA) and then DNA fragments were enriched using SureSelect Target Enrichment Kit (Agilent technologies, Inc., USA). Several libraries were pooled, and then bridge amplification on cBot (Illumina, Inc., San Diego, CA). Finally, the sequencing was performed with an Illumina HiSeq Sequencer (illumine, Inc., San Diego, CA) using the 2 × 150 bps paired-end read module to get the FastQ data.

### In silico analysis

The FastQ sequence reads were aligned to the human genome reference sequence (hg19) using the Barrows-Wheeler Aligner (BWA) for analysis. The variants with low coverage depth were excluded for further analysis. The called SNVs/InDEL with high quality were annotated using Annovar program. We defined a “novel variant” if: 1) it had no rsID; 2) it has not been recorded in the public database including Human Gene Mutations Database (HGMD) and ClinVar. For the novel variants, PolyPhen-2, Sorting Tolerant From Intolerant (SIFT) and MutationTaster were used to predict the pathogenicity of them. Combined Annotation Dependent Depletion (CADD), Dann and Eigen were used to assess the deleteriousness of insertion/deletions variants. Furthermore, the novel pathological variants were confirmed by Sanger sequencing. Based on HGVS nomenclature, a “null” LDLR mutation referred to the nonsense, frameshift and large rearrangements while a “defective” LDLR mutation was pathogenic point mutations.

### Statistical analysis

All the statistical analysis was performed using SPSS version 21.0 (SPSS Inc., Chicago Illinois, USA). Continuous variables with normal distribution were presented as mean ± SD and median (Q1–Q3 quartiles) represented continuous but with non-normal distribution variables. Categorical variables were presented as number (percentage). To compare the differences among groups, continuous parameters were analyzed with Student’ s *t*-test, analysis of variance (ANOVA) or Mann-Whitney U test. Categorical variables were analyzed using chi-square test and Fisher’s exact test if applicable. A *p*-value < 0.05 was considered significantly different.

## Results

### Patient characteristics

Baseline characteristics of the cohort were shown in Table 1. The mean age of the subjects was 49 ± 12 years old and 61.1% (*n* = 174) were men. Patients came from all over China while the majority were from northern China. Additionally, a total of 28 patients (9.8%) presented with xanthoma but 81.8% of the subjects had CAD. The average level of TC was 7.03 ± 2.53 mmol/L and LDL-C was 5.22 ± 2.12 mmol/L at enrollment. The majority of the participants (81.1%) were treated with statins and 6.0% were treated with ezetimibe (with statin or alone). The well documented and estimated average level of untreated LDL-C was 7.86 ± 2.25 mmol/L.

**Table 1:** Clinical characteristics of the unrelated index cases

Characteristics	FH patients (*n* = 285)
Baseline Data	
Male, n (%)	173 (60.7%)
Age, year	49 ± 12
BMI, kg/(m^2^)	25.32 ± 3.74
SBP, mm/Hg	125 ± 17.0
DBP, mm/Hg	78 ± 11.5
Glucose, mmol/L	5.54 ± 1.47
Xanthoma, n(%)	28 (9.8%)
CAD, n (%)	233 (81.8%)
Family history of CAD, n (%)	124 (43.5%)
Region distribution	
northeast China	65 (22.8%)
north China	160 (56.1%)
northwest China	12 (4.2%)
eastern China	38 (13.3%)
southern China	8 (2.8%)
southwest China	2 (0.7%)
Lipids	
TC, mmol/L	7.03 ± 2.53
HDL-C, mmol/L	1.09 ± 0.32
LDL-C, mmol/L	5.22 ± 2.12
Untreated LDL-C, mmol/L	7.86 ± 2.25
TG, mmol/L	1.67 (1.20–2.14)
ApoA, g/L	1.31 ± 0.33
ApoB, g/L	1.47 ± 0.49
Statin, n (%)	231 (81.1%)
Ezetimibe, n (%)	17 (6.0%)

Data are expressed as mean ± SD, median (25th–75th percentile) or n (%)

*FH* familial hypercholesterolemia, *BMI* body mass index, *SBP* systolic blood pressure, *DBP* diastolic blood pressure, *CAD* coronary artery disease, *TC* total cholesterol, *HDL-C* HDL cholesterol, *LDL-C* LDL cholesterol, *TG* triglyceride, *apo A* apolipoprotein A, *apo B* apolipoprotein B

### Mutation analysis

Overall, we sequenced the LDLR, APOB and PCSK9 genes and identified 137 distinct variants altogether. However, 18 variants without report were predicted to be benign. Thus, we identified 119 distinct risk variants in 148 patients with a detection rate 51.9%. in other word, in 137 patients with a clinical diagnosis of FH, we did not find a mutation. Of the 148 patients with a positive mutation, 77 patients were LDLR heterozygotes (25 with null mutation and 52 with defective mutations, respectively), 33 were APOB mutation carriers and 4 were PCSK9 carriers. Furthermore, 13 double heterozygotes, 16 compound heterozygotes and 5 true LDLR homozygotes were also identified.

**Fig. 1:**
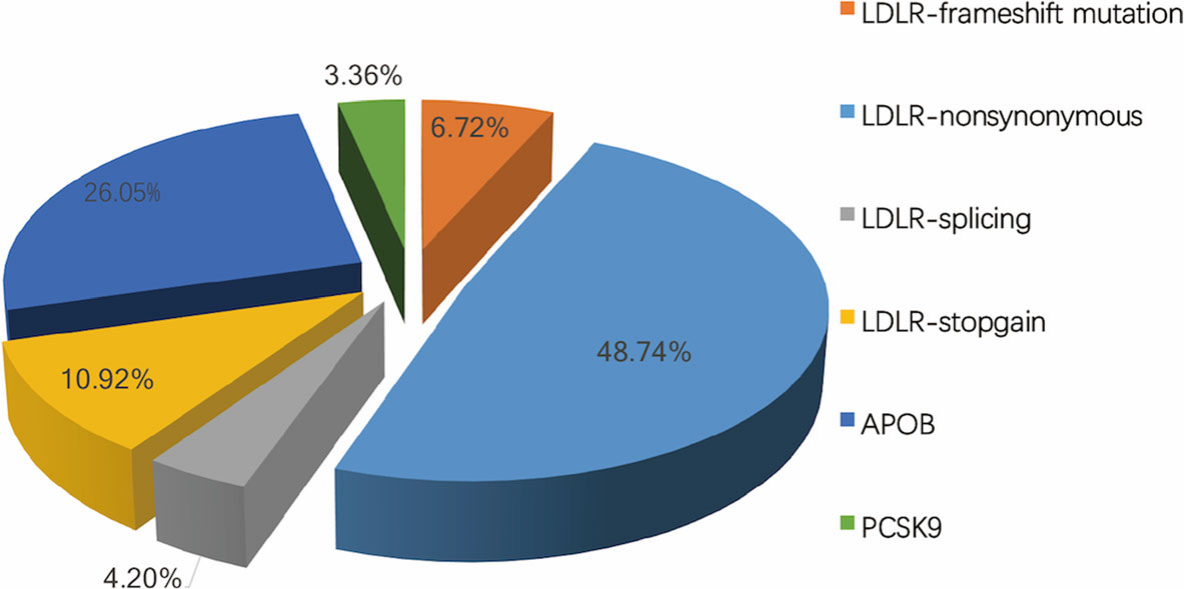
Distribution of different types of all the FH-related variances (*n* = 119) detected in the study. FH: familial hypercholesterolemia; LDLR: low-density lipoprotein receptor; APOB: apolipoprotein B; PCSK9: proprotein convertase subtilisin/Kexin type 9

The distribution of types of the 119 risk variants was shown in Fig. 1**.** In detail, of the 119 distinct variants, 84 were in LDLR gene accounting for 70.59% with 58 nonsynonymous mutations, 8 frameshift mutations, 5 splicing and 13 stopgain mutations. The APOB and PCSK9 variants accounted for 26.05% (31/119) and 3.36% (4/119) respectively. Twenty-eight variants were found in more than one individual and LDLR c.1448G > A (p. W483X) was most frequent one detected in 9 patients. The variants of LDLR distributed on a total of 17 exons with the most frequent ones on exon 4 (*n* = 19) while APOB variants appeared on the exon 26 most (*n* = 13) but we also detected variants on other 13 exons.

Besides, we found 8 (7 LDLR and 1 APOB) novel variants referred as “pathogenic variants” according to in silico analysis shown in Table 2. There were two frameshift deletion mutations on the LDLR gene and five nonsynonymous mutations. The only one APOB novel variant was located on exon 26 and was a nonsynonymous one.

**Table 2:** Novel potential pathogenic variants identified in the study

Gene	Exon	DNA change	Protein change	Function	Number	Prediction			ACMG classification
						PolyPhen	SIFT	Mutation Taster	
LDLR									
3		c.285C > G	p.C95W	nonsynonymous	1	probably damaging	damaging	disease causing	PM2;PM5;PP3
3		c.302A > T	p.E101V	nonsynonymous	1	probably damaging	damaging	disease causing	PM2;PP3
4		c.393delC	p.D131fs	frameshift deletion	1	NA	NA	NA	PVS;PM2
4		c.524A > G	p.D175G	nonsynonymous	1	probably damaging	tolerated	disease causing	PM2
5		c.728G > A	p.C243Y	nonsynonymous	1	probably damaging	damaging	disease causing	PM2;PP3
9		c.1206delC	p.F402 fs	frameshift deletion	1	NA	NA	NA	PVS;PM2
13		c.1934A > T	p.N645I	nonsynonymous	1	possibly damaging	damaging	disease causing	PM2;PP3
APOB									
26		c.10093C > G	p.H3365D	nonsynonymous	1	probably damaging	damaging	polymorphism	PM2

*NA* not applicable, *LDLR* low-density lipoprotein receptor, *APOB* apolipoprotein B

Furthermore, we have listed the most common LDLR mutations found in the current study and shown their geographic distributions in Table 3. Three mutations only distributed in the northern regions (c.769C > T; c.1765G > A; c.1864G > T) and the splicing mutation (c.1187-10G > A) was mainly located in the east. The other three mutations (c.1448G > A; c.1879G > A; c.1747C > T) distributed both in the north and south.

**Table 3:** The geographic distribution of the most common LDLR mutations of FH in China

DNA change	Mutation	Number	Geographic distribution (number)					
			Northeast China	North China	Northwest China	Eastern China	Southern China	Southwest China
c.1448G > A	p.W483X	9	0	3	0	2	3	1
c.1879G > A	p.A627T	7	2	2	0	2	1	0
c.769C > T	p.R257W	5	1	3	1	0	0	0
c.1765G > A	p.D589N	5	1	3	1	0	0	0
c.1187-10G > A		4	1	0	0	3	0	0
c.1747C > T	p.H583Y	4	2	1	0	0	1	0
c.1864G > T	p.D622Y	4	0	3	1	0	0	0

*LDLR* low-density lipoprotein receptor, *FH* familial hypercholesterolemia

### Genotype-phenotype correlation

To correlate genotype to phenotype, we compared the clinical characteristics, especially lipid levels, between index cases with different genotypes. Patients carrying LDLR null mutations showed significantly higher lipid levels compared with those carrying LDLR defective mutations, APOB/PCSK9 mutations as well as no mutations (Table 4). Of note, we compared the untreated LDL-C level and found the same results though the group of LDLR null mutations carriers received significantly fewer lipid lowering treatment.

**Table 4:** Characteristics of index cases with FH according to the genotype

Variables	Mutation (−) (*n* = 137)	Mutation (+) (*n* = 112)			*p*-value
		LDLR null heterozygote (*n* = 25)	LDLR defective heterozygote (*n* = 52)	APOB/PCSK9 mutation (*n* = 37)	
Age, year	51 ± 10	50 ± 12	47 ± 14	47 ± 14	0.099
Male, n (%)	89 (65%)	16 (64%)	30 (57.7%)	20 (54.1%)	0.584
Xanthoma, n (%)	5 (3.6%)	1 (4%)	11 (21.2%)	2 (5.4%)	**0.001**
CAD, n (%)	114 (83.8%)	21 (84%)	42 (80.8%)	32 (86.5%)	0.911
Family history of CAD, n (%)	62 (45.6%)	12 (48%)	23 (46%)	15 (40.5%)	0.935
Statin, n (%)	123 (89.8%)	17 (68%)	38 (73.1%)	32 (86.5%)	**0.005**
TC, mmol/L	6.51 ± 1.97	7.88 ± 1.55	7.14 ± 2.35	6.46 ± 2.06	**0.007**
HDL-C, mmol/L	1.15 ± 0.3	1.05 ± 0.29	1.05 ± 0.35	1.09 ± 0.33	0.138
LDL-C, mmol/L	4.63 ± 1.5	6.22 ± 1.48	5.47 ± 2.03	4.7 ± 1.5	**< 0.001**
Untreated LDL-C, mmol/L	7.5 ± 1.82	8.88 ± 1.82	7.94 ± 2.13	7.39 ± 1.56	**0.004**
Apo A, g/L	1.4 ± 0.34	1.2 ± 0.26	1.23 ± 0.27	1.35 ± 0.34	**0.002**
Apo B, g/L	1.37 ± 0.38	1.64 ± 0.37	1.55 ± 0.52	1.35 ± 0.41	**0.003**
TG, mmol/L	1.72 (1.26–2.28)	1.45 (1.18–1.88)	1.59 (1.07–1.88)	1.78 (1.51–2.14)	**0.032**

Data are expressed as mean ± SD, median (25th–75th percentile) or n (%). Bold values indicate statistical significance

*FH* familial hypercholesterolemia, *LDLR* low-density lipoprotein receptor, *APOB* apolipoprotein B, *PCSK9* proprotein convertase subtilisin/Kexin type 9, *CAD* coronary artery disease, *TC* total cholesterol, *HDL-C* HDL cholesterol, *LDL-C* LDL cholesterol, *apo A* apolipoprotein A, *apo B* apolipoprotein B, *TG* triglyceride

**Fig. 2:**
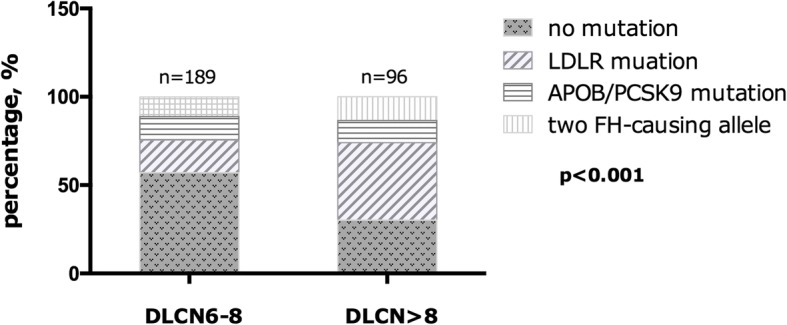
Comparison of FH-related mutations according to DLCN score. FH: familial hypercholesterolemia; DLCN: Dutch Lipid Clinic Network; LDLR: low-density lipoprotein receptor; APOB: apolipoprotein B; PCSK9: proprotein convertase subtilisin/Kexin type 9

As for the homozygote in regard of genotype, the 5 patients with true LDLR homozygous mutations showed the most severe phenotype at younger age (23 ± 9 vs. 42 ± 14 and 46 ± 12, respectively). They present much more xanthoma and higher level of TC and LDL-C but significantly lower level of HDL-C. Without doubt, double heterozygotes showed a much milder phenotype compared to the compound and true homozygotes (Table 5). Besides, the mutation spectrum was significantly different between patients with DLCN 6–8 score group and DLCN > 8 score group (Fig. 2). The latter had higher positive mutation detection rate with more LDLR and two FH-causing mutations (*p* < 0.05).

**Table 5:** Characteristics of carriers with more than one FH causing variances

	Double heterozygotes^a^	LDLR compound heterozygotes	LDLR true homozygotes	*p*-value
	*n* = 13	*n* = 16	*n* = 5	
Age, year	46 ± 12	42 ± 14	23 ± 9	**0.006**
Male, n (%)	6 (46.2%)	10 (62.5%)	2 (40%)	0.580
Xanthoma, n(%)	1 (7.7%)	4 (25%)	4 (80%)	**0.011**
CAD, n (%)	8 (61.5%)	13 (81.3%)	3 (60%)	0.510
Statin, n(%)	8 (61.5%)	12 (75%)	1 (20%)	0.124
Family history of CAD, n (%)	4 (30.8%)	7 (43.8%)	1 (25%)	0.692
TC, mmol/L	7.19 ± 2.11	8.25 ± 3.77	16.05 ± 2.94	**< 0.001**
HDL-C, mmol/L	1.1 ± 0.33	0.92 ± 0.19	0.59 ± 0.53	**0.028**
LDL-C, mmol/L	5.51 ± 1.71	6.51 ± 3.3	12.68 ± 2.94	**< 0.001**
LDL-C range, mmol/L	3.05–8.86	3.31–15.78	9–15.96	
Untreated LDL-C, mmol/L	6.89 ± 1.12	9.03 ± 3.85	14.34 ± 3.77	**< 0.001**
Apo A, g/L	1.21 ± 0.25	1.12 ± 0.31	0.45 ± 0.28	**0.007**
Apo B, g/L	1.59 ± 0.66	1.86 ± 0.91	2.7 ± 0.3	0.199
TG, mmol/L	1.29 (0.99–2.12)	1.49 (1.07–2.08)	0.9 (0.77–1.63)	0.357

Data are expressed as mean ± SD, median (25th–75th percentile), or n (%). Bold values indicate statistical significance

*FH* familial hypercholesterolemia, *CAD* coronary artery disease, *TC* total cholesterol, *HDL-C* HDL cholesterol, *LDL-C* LDL cholesterol, *apo A* apolipoprotein A, *apo B* apolipoprotein B, *TG* triglyceride

^a^a double heterozygote was carriers of LDLR+APOB or LDLR+PCSK9

## Discussion

In this genetic study, we screened 285 unrelated index cases with clinical definite/probable FH in the three FH-causing genes LDLR, APOB and PCSK9 using NGS. Overall, we got a FH mutation detection rate of 51.9% and found 8 novel variants response for FH in such unrelated index cases. Furthermore, our study also confirmed the genotype –phenotype correlations of FH. To our knowledge, this has been the largest cohort to characterize the genetic spectrum of Chinese population originating from almost the whole mainland China so far.

The detection rate of FH-causing mutations varies depending on the ethnic groups, screening method and diagnostic criteria. In the current study, we identified a positive mutation in 51.9% of the 285 unrelated probands diagnosed with DLCN criteria, which was similar to the previous studies conducted in Singapore, European and Brazilian populations [21–23]. With the deepening understanding of genetic basis of FH, studies have demonstrated that the marked elevation of LDL-C was due to far more than the traditional “monogenic FH”. Other reasons that lead to the occurrence of FH includes rare mutations in known FH genes, mutations in a novel gene, polygenic FH secondary to the cumulative effect of LDL-C raising single nucleotide polymorphisms (SNP), and other acquired phenocopies [24, 25]. Besides, mutations in the gene ABCG5 and ABCG8 could cause sitosterolemia, which may lead to misdiagnosis of FH because of similar phenotypes [26].

Across nations, the molecular spectrum of FH differs significantly. In some European countries, a founder effect exists with the predomination of a few mutations because of the relatively univocal population [27, 28]. While a previous study from central south region of China found only 43 mutations in 219 FH patients [19], the present study identified a total of 119 FH-associated variants in 285 patients and found that the variant LDLR c.1448G > A (p. W483X) presented the highest frequency but with only 9 carriers. The data suggested a high genetic heterogeneity for FH in Chinese population which may attribute to the boarder geographical regions and nationalities. Similar to the previous studies, we also found that mutations in the LDLR gene made up the vast majority and most variants of LDLR located in the exon 4. A systematic review concluded that c.1879G > A, c.1448G > A and c.1864G > T were the primary LDLR mutations in 295 probands from mainland china, which were consistent with the present study [29]. Also, the mutation LDLR c.1747C > T reported most in southern China, Taiwan and Singapore was also common in the patients from northern China in our study [30]. Interestingly, after analyzing the genotype of the compound heterozygotes, we found that 4 probands carried the same alleles: LDLR c.769 C > T(p.R257W) + LDLR c.1765 G > A(p.D589N). Nevertheless, we have also identified the well-known APOB mutation c.10579C > T(p.R3527W) and c.10580G > A(p.R3527Q) but in only one patient respectively. The most common FH-associated APOB variant in the current population was c.4163G > A(p.R1388H), which was previously reported in Malaysia [31]. In fact, we found variants in other 13 exons besides exon 26, the most well know mutation cluster in APOB gene, thus the sequencing should cover the entire gene. Furthermore, we also detected 4 distinct variants in the PCSK9 gene which needed more functional studies with less descriptions before in Chinese population.

Nine novel “pathogenic” or “likely pathogenic” mutations have been identified in this study with seven in LDLR gene and one in APOB gene. In detail, five novel mutations are encoded in the ligand binding domain of LDLR, which is important for the binding of LDL to the receptor [32]. Another two novel mutations are located in the EGF-like domain of LDLR and may affect the receptor dissociation in endocytosis and recycling to the cell surface [32]. The APOB mutation was located in the exon 26, the proposed LDL-receptor-binding domain, and may affect the process of endocytosis [33].

Not surprisingly, the severity of phenotype varies across the genotype. Mounting evidence have demonstrated that carriers of LDLR mutation, especially those with LDLR null mutation, had the highest lipid levels in patients with heterozygous FH, which is in agreement with the present study [34, 35]. Furthermore, the phenotype of homozygous and compound heterozygous LDLR mutation carriers overlaps to a large extent with worse manifestations. But the double heterozygotes, usually combination of LDLR and APOB/PCSK9 mutation, are suspected to have an intermediate phenotype because of the milder phenotype of APOB/PCSK9 carriers [36, 37]. In the current study, we found that patients with double heterozygous FH had relatively lower lipid levels. Of note, the representation of the date may be weakened by the small sample size.

There are limitations in our study. First, despite the greatest cohort from multiple centers in mainland China at present, it still cannot represent the precise genetic spectrum of Chinese population because of the unawareness and underdiagnoses of FH in China. Second, partial untreated LDL-C levels were not available and we estimated them by correction factors according to treatment potency [38]. Third, we were not able to perform co-segregation and functional analysis in patients with novel variants.

## Conclusion

In summary, the current study replenished the knowledge of mutation spectrum of FH in China and further confirmed the heterogeneity of FH genetics and genotype-phenotype correlations in Chinese population. Data could help design a nationwide future screening plan to fill the gap of genetic basis of FH in China and further promote early screening and disease management.

## Abbreviations

APOB: Apolipoprotein B
ASCVD: Atherosclerotic cardiovascular disease
CAD: Coronary artery disease
DLCN: Dutch Lipid Clinic Network
FH: Familial hypercholesterolemia
HDL-C: High-density lipoprotein cholesterol
LDL-C: Low-density lipoprotein cholesterol
LDLR: Low-density lipoprotein receptor
NGS: Next generation sequencing
PCSK9: Proprotein convertase subtilisin /kexin type 9
TC: Total cholesterol
TG: Triglyceride
